# Effects of Aging on Hair Color, Melanosome Morphology, and Melanin Composition in Japanese Females

**DOI:** 10.3390/ijms20153739

**Published:** 2019-07-31

**Authors:** Takashi Itou, Shosuke Ito, Kazumasa Wakamatsu

**Affiliations:** 1 Kao Corporation, R&D - Hair Care Products Research, Tokyo 131-8501, Japan; 2 Department of Chemistry, Fujita Health University School of Medical Sciences, Toyoake, Aichi 470-1192, Japan

**Keywords:** hair, aging, melanosome, morphology, melanin, 5,6-dihydroxyindole, 5,6-dihydroxyindole-2-carboxylic acid

## Abstract

In a previous study, we showed that the size of melanosomes isolated from Japanese female hairs enlarges with age, and this affects the hair color. In this study, we analyzed the age-dependent changes in hair melanin in order to further explore the factors related to hair color changing by aging. A significant positive correlation with age was found in the total melanin amount (TM) and the mol% of 5,6-dihydroxyindole (DHI) units, while no correlation was found in pheomelanin mol%. TM showed significant correlations with hair color parameters and the melanosome volume, suggesting that hair color darkening by aging is caused by the increase in TM due to the enlargement of the size of melanosome. From the measurement of absorbance spectra on synthetic eumelanins with different ratios of DHI and 5,6-dihydroxyindole-2-carboxylic acid (DHICA), we found that the increase in DHI mol% also contributes to the darkening of hair color by aging. In addition, the level of pyrrole-2,3-dicarboxylic acid (PDCA), a marker of DHI melanin, showed a significant negative correlation with the aspect ratio of melanosome, suggesting a contribution of DHI melanin to the change in melanosome morphology by aging.

## 1. Introduction

Human scalp hair contains melanins, which have important roles such as the protection of the head from harmful ultraviolet (UV) light, the elimination of toxic heavy metals and chemicals [1], and the attractive appearance. Melanins are produced in melanocytes in hair follicles. Graying is the most drastic change by aging in human hair, and its causes have been well studied regarding a decrease in the number of melanocytes and a depletion of melanocyte stem cells in hair follicles [2,3]. Research on the causes of hair color is important in forensic science, and the relationship between genes and hair color has recently been studied [4,5,6]. For blonde hair, it is known that the darkening of hair color occurs as a child grows up; however, the reason for this has not been clarified [7]. There are not many studies related to hair color changing with age apart from hair graying.

Hair color mainly depends on the quantity, the quality, and the distribution of the pigment melanin [8]. There are two types of melanin, black to brown eumelanin and yellow to reddish-brown pheomelanin, and their biosynthesis pathways are well known [8]. Eumelanin consists of two kinds of moiety; one is from 5,6-dihydroxyindole (DHI), and the other is from 5,6-dihydroxyindole-2-carboxylic acid (DHICA). DHICA melanin exhibits a potent hydroxyl radical-scavenging activity, whereas DHI melanin does not [9]. It was proposed that the formation of weak aggregates accounts for a greater accessibility of free radicals to DHICA compared to the compact π-stacked DHI melanin [10].

Commo et al. [11] found that the hair tone of East Asian (Chinese) and Caucasian hair became darker with age, and that their total melanin amount (TM) increased with age. They also analyzed contents of pyrrole-2,3-dicarboxylic acid (PDCA) and pyrrole-2,3,5-tricarboxylic acid (PTCA) and found that the ratio of PDCA/PTCA was significantly dependent on age for Caucasian and African American hairs, meaning that an eumelanin component, DHICA mol%, changed by aging, while the correlation was not significant for East Asian hairs. Ito et al. [12] proposed the method to quantify pheomelanin content more accurately by treating hairs with hot HCl to remove protein components from hair prior to an alkaline hydrogen peroxide (H_2_O_2_) oxidation. They found a constant level of pheomelanin in human black to brown hairs. 

The color of Japanese female hair is generally thought to be black, but it was found that the color changes with age [13]. A significant age dependence on the morphology of hair melanosomes was also found. The minor axis of isolated melanosomes significantly increased, and as a consequence, the aspect ratio (major/minor axes) significantly decreased with age. These morphological parameters showed significant correlations with the hair color, although the subject number used for the correlation analysis was only 10. In addition to that, the hair melanin, which directly relates to the hair color, was not analyzed in the previous study.

In this study, we analyzed the hair melanin of Japanese females (*n* = 33) of a wide age range in order to explore the relationship among age, hair color, melanosome morphology, and melanin composition.

## 2. Results

All results including the previous study [13] are summarized in Tables S1 and S2. Missing data indicate that the sample amount was too small for the experiment.

### 2.1. Age Dependence of Hair Color

**Figure 1:**
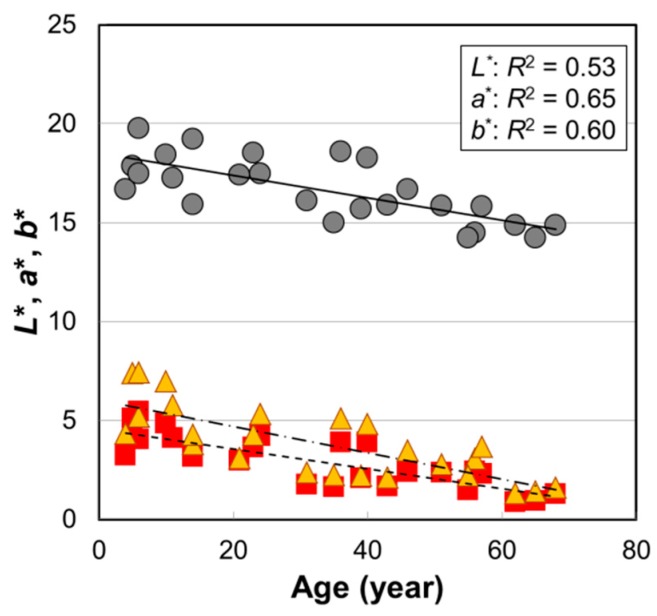
The age dependences of hair color parameters L^*^, a^*^, and b^*^ values of Japanese female hair bundles. Circles, L^*^; squares, a^*^; triangles, b^*^. Lines are the fitted curves. Solid line, L^*^; dashed line, a^*^; dot-and-dash line, b^*^. *p* = 3.3 × 10^−5^ (L^*^), 1.1 × 10^−6^ (a^*^), and 5.6 × 10^−6^ (b^*^).

Figure 1 shows the age dependence of hair color parameters L^*^, a^*^, and b^*^ from the ages 4–68 years old (*n* = 25). These values significantly decrease with age (Table 1). The age dependence of the cross-sectional area for these hair fibers showed a maximum at around the 30–40’s (Figure S1), which was the same trend as reported previously [14] and indicated that the hair color had no correlation with the cross-sectional area of the hair fiber (Figure S2).

Similar to the case of Caucasian hair [7], it is generally recognized for Japanese hair that the hair color is sometimes brown in childhood but turns darker as children grow up. However, there was no information about how the color of pigmented hair changes after maturity. Commo et al. [11] reported a significant age dependence of hair tone level—assessed using a light color scale of 10 steps—not only in Caucasian hair (*R*^2^ = 0.18, *p* = 0.0012) but also in East Asian (Chinese) hair (*R*^2^ = 0.097, *p* = 0.020). In this study, for pigmented hairs of Japanese females, we found higher correlations with age (*R*^2^ = 0.53, *p* = 3.3 × 10^−5^ for L^*^, *R*^2^ = 0.65, *p* = 1.1 × 10^−6^ for a^*^, and *R*^2^ = 0.60, *p* = 5.6 × 10^−6^ for b^*^), and it was clearly seen that the hair color continued to be darker with age after pubertal age.

### 2.2. Melanosome Morphology

Most of the isolated melanosomes were seen to be ellipsoidal forms, as in the previous study [13], while their shape was changed from slender ellipsoid to a thicker shape with age (Figure S3). We determined the major and the minor axes, the aspect ratio, and the volume of isolated melanosomes in an increased number of subjects (*n* = 38) compared to the previous study (*n* = 24; [13]). We examined the correlation analyses of obtained morphological parameters with age, including those from the previous study (Figure S4). The minor axis significantly increased with age (*R*^2^ = 0.36, *p* = 6.0 × 10^−5^), while there was no age dependence in the major axis. The correlation of the aspect ratio with age was the highest (*R*^2^ = 0.61, *p* = 6.1 × 10^−9^), showing that the ellipsoidal shape of melanosome changed to be broad with age. The mean volume of melanosome *V* estimated with an assumption of ellipsoid had large individual variation, and some data points were out of the correlation line; however, it was confirmed that *V* also significantly increased with age (*R*^2^ = 0.21, *p* = 0.0039).

We also examined the correlations between the hair color and the morphology parameters of melanosome determined above (Figure S5). In the previous study, significant correlations were obtained only with the minor axis and the aspect ratio. However, in addition to these, melanosome volume also showed a significant correlation (*p* < 0.050) with the hair color parameters, L^*^ and b^*^ (Table 1), owing to more data points (*n* = 25) than the previous study (*n* = 10).

### 2.3. Absorption Spectra of Eumelanins Synthesized with DHI and DHICA

Absorbance at 500 nm (A500) is an indicator of TM, while the ratio of absorbance at 650 nm (A650/A500) reflects mol% of pheomelanin, pure pheomelanin giving a value of 0.1 and pure eumelanin giving a value of > 0.3 [15]. In the present study, the effect of DHI/DHICA ratio on the A650/A500 ratio was first examined.

**Figure 2:**
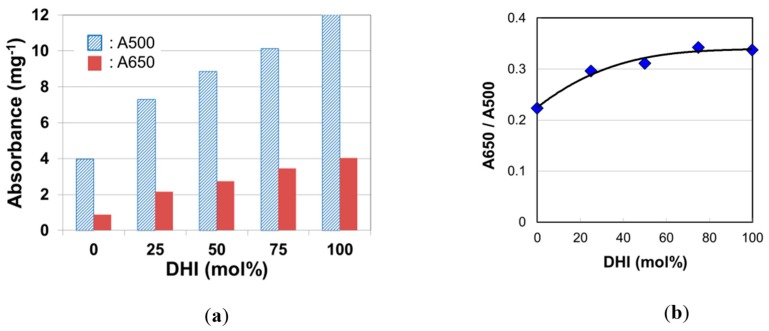
(**a**) The absorbance at the wave length of 500 nm (A500) and 500 nm (A650) of Soluene-350 solutions of melanins synthesized with various 5,6-dihydroxyindole (DHI) mol%. (**b**) Effect of DHI mol% on the absorbance ratio A650/A500. The data are averages from two independent analyses.

Panzella et al. [10] measured UV-visible spectra on dispersions of synthetic DHI melanin and DHICA melanin and found that the spectra of the two melanins were quite different. There may have been an issue in the difference in solubility of both melanins, but the difference was observed in the absorbance in the range of visible light. In our study, to examine the effect of DHI mol% on hair color, we measured absorption spectra of solubilized melanins synthesized with DHI, DHICA, and their mixtures in various mol% (Figure S6). The absorbance of DHI melanin was higher than that of DHICA melanin in the wavelength range from 400 to 800 nm. Figure 2 displays trends of A500, A650, and A650/A500 as DHI mol% increased. Both A500 and A650 increased with DHI mol% (Figure 2a), and it was found that their ratio, A650/A500, also increased with DHI mol% (Figure 2b).

### 2.4. Melanin Compositions

**Figure 3:**
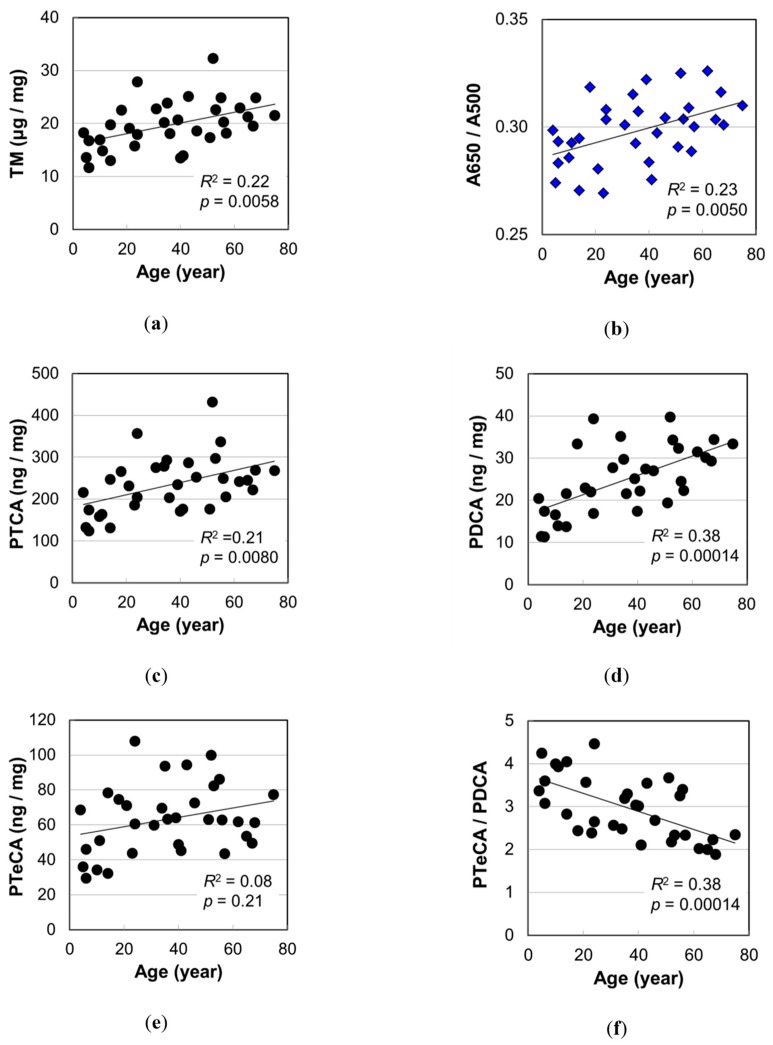
The age dependencies of various values measured by melanin analyses of Japanese female hairs. (**a**) Total melanin amount (TM). (**b**) Absorbance ratio A650/A500 for Soluene-350 solution of hair melanin. (**c**) Level of pyrrole-2,3,5-tricarboxylic acid (PTCA) in hair. (**d**) Level of pyrrole-2,3-dicarboxylic acid (PDCA) in hair. (**e**) Level of pyrrole-2,3,4,5-tetracarboxylic acid (PTeCA) in hair. (**f**) The ratio PTeCA/PDCA. The coefficient of determination and *p*-value are shown in each figure.

Melanin analysis was performed on 33 hair samples. The results are summarized in Table S2, and the age dependencies are illustrated in Figure 3. TM evaluated spectrophotometrically by analyzing A500 after solubilization of melanin in Soluene-350 and the ratio of absorbance A650/A500 are shown in Figure 3a,b, respectively. Significant positive correlations between TM and age (*p* = 0.0058) and between A650/A500 and age (*p* = 0.0050) were observed, suggesting that the hair color turned darker and the color shade changed with age.

PTCA, PDCA, and pyrrole-2,3,4,5-tetracarboxylic acid (PTeCA) serve as markers of DHICA melanin, DHI melanin, and cross-linked DHI melanin, respectively [16,17,18]. The levels of eumelanin makers determined by high performance liquid chromatography (HPLC) after alkaline H_2_O_2_ oxidation, PTCA, PDCA, and PTeCA are plotted against age in Figure 3c–e, respectively. Among these three markers, PDCA level showed the highest correlation with age (PTCA, *p* = 0.0080; PDCA, *p* = 0.00014; PTeCA, *p* = 0.21). On the other hand, PTeCA level did not show significant correlation with age (Figure 3e), thus the ratio of cross-linking to non-cross-linking DHI components, corresponding to PTeCA/PDCA, showed a significant negative correlation with age (*p* = 0.00014) (Figure 3f). This result is reasonable because a cross-linking becomes difficult to form by the increase in DHI mol% with age.

The cross-linking formation of melanin occurs by an environmental stress, such as heat after hair fiber generation, in addition to the formation during melanogenesis in the follicle. In the case of posteriori cross-linking formation, it is necessary that segments are approached with each other by the molecular motion. The structure of the DHI melanin segment takes an advanced planar association structure by intermolecular π-stacking, while the DHICA melanin segment takes a non-planar structure with a weak intermolecular interaction [10]. From the structural characteristics, the DHI melanin segment has low molecular mobility, and it seems difficult to form an acquired cross-linkage at both C-2 and C-3 positions. Conversely, the DHICA melanin segment has higher mobility, and it is expected to be more easily cross-linked by heating. To prove this, however, it is necessary to verify by comparing the PTeCA amount before and after giving the same heat treatment to hair samples having different ratios of DHI and DHICA.

**Figure 4:**
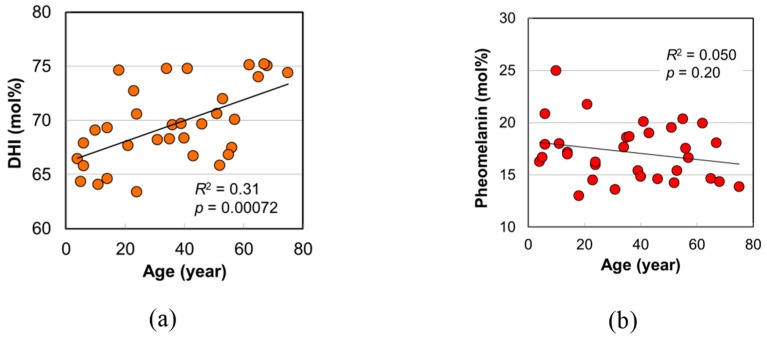
The age dependences of melanin components in Japanese female hairs. (**a**) DHI mol% in eumelanin of hair. (**b**) Pheomelanin mol% in hair. The coefficient of determination and the *p*-value are shown in each figure.

The DHI mol% in eumelanin was determined from the ratio of PDCA/PTCA. In addition, the pheomelanin mol% was obtained from the ratio of thiazole-4,5-dicarboxylic acid (TDCA) to PDCA evaluated through the acid hydrolytic treatment of hair by HCl followed by alkaline H_2_O_2_ oxidation and HPLC analysis [12]. The DHI mol% and the pheomelanin mol% are plotted against age in Figure 4a,b, respectively. A significant positive age dependence of DHI mol% was found (*p* = 0.00072). On the other hand, pheomelanin mol% had no correlation with age. The obtained values of pheomelanin mol% were in the range of 13 to 25%, and their mean value was 17.2 ± 2.6%, which was consistent with the result for East Asian hair (17.0%) determined recently [12].

From trace mineral analyses, it was shown that the copper ion (II) concentration in female hair decreased with age [19] or decreased after 40 years of age [20], although the statistical significance was not found. These were the results of analyzing trace elements in hair shafts, and the age dependence of copper ion (II) concentration in hair follicles and melanocytes is still unknown. However, the decrease in copper ion (II) by aging might be the cause of the increase in DHI mol% with age, since copper ion has been shown to promote the conversion of dopachrome to DHICA [21]. The melanin composition should be affected by the expression levels of tyrosinase, tyrosinase-related protein-1, and dopachrome tautomerase, and their ratios; however, there is no information thus far about the age dependence of them as far as we know.

The tendency that DHICA mol% decreases by aging has been found thus far. According to Commo et al. [11], the DHICA mol% in African-American hair was 50% in the group of children under 11 years old and 33% in the group of adults over 46 years old. In Caucasian hair, it decreased from 58% to 45% as well. In the case of black East Asian hair, however, it was 33%, and the age dependence was not found.

Regarding the cause of the discrepancy in the results on black East Asian hair, it may be noted that the hair samples used by Commo et al. were limited up to 65 years old. According to Figure 4a, the DHI mol% was high for the elderly subjects over 65 years old, and these data points seem to contribute greatly to the age dependence. When we examined the analysis (except for these data points), the correlation of the DHI mol% with age became quite low but still had some correlation (*R*^2^ = 0.13, *p* = 0.050). As for another cause, hair fibers were shredded in the melanin analysis by Commo et al., whereas we used the homogenized hair in this study, a method by which the variation of the data is expected to be smaller [16]. Furthermore, we used more weight of hair samples in the melanin analysis than Commo et al. As described above, from the wider age range of subjects and the higher accuracy of the experiment, this study supplemented the previous results, and it was clarified that DHICA mol% decreased by aging, regardless of the ethnic origin.

It is known that DHI melanin and DHICA melanin are different in the association structure and the strength of the intermolecular interaction and that DHICA melanin has better ability in regards to the free-radical-scavenging property [10], which is one of the important facets of melanin existence in living organisms. The change in melanin composition found this time would be a new aging phenomenon of hair.

The DHI mol% obtained in this study for Japanese female hair existed in the range from 63% to 75% (Figure 4a). According to Figure 2b, the A650/A500 value in the DHI mol% range (63–75%) was almost constant at about 0.33, which was higher than the values of actual hair melanin (0.27–0.33) (Figure 3b). This discrepancy may have come from the fact that actual hairs have small amounts (ca. 15%) of pheomelanin [12] and have undergone some UV light decomposition, thereby affecting A650/A500 [22]. As a whole trend of relative change, it became clear that the A650/A500 value increased with DHI mol%.

**Figure 5:**
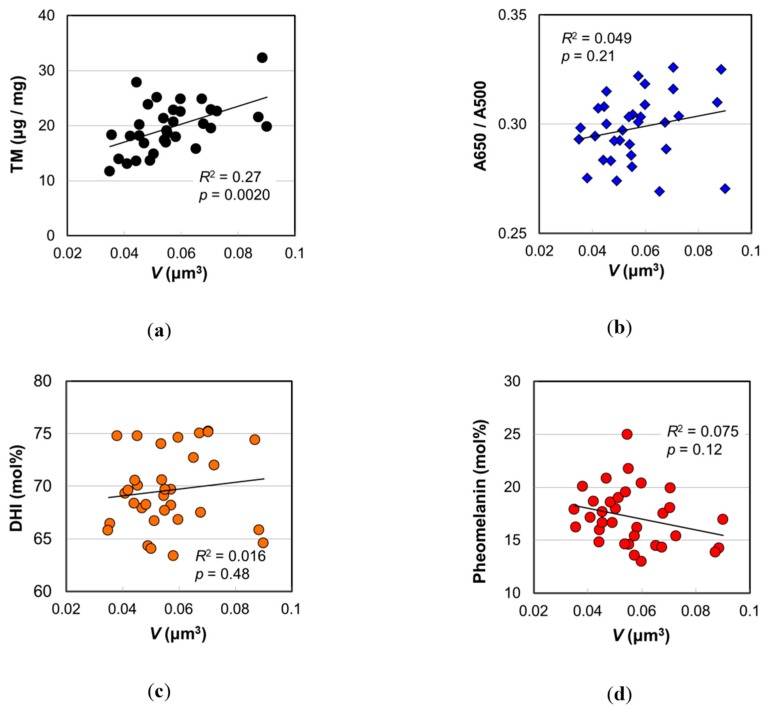
Various values measured by hair melanin analyses vs. mean volume of melanosomes isolated from Japanese female hair samples with an assumption of ellipsoid. (**a**) Total melanin amount. (**b**) Absorbance ratio A650/A500. (**c**) DHI mol%. (**d**) Pheomelanin mol%. The coefficient of determination and the *p*-value are shown in each figure.

Figure 5 shows the correlations between the values determined by the melanin analyses and the mean melanosome volume. It was confirmed that TM was significantly higher as *V* became larger (Figure 5a) (*R* ^2^ = 0.27, *p* = 0.0020). On the other hand, there were no significant correlations between *V* and A650/A500 (Figure 5b), DHI mol% (Figure 5c), and pheomelanin mol% (Figure 5d). It should be noted that A650/A500, DHI mol%, and pheomelanin mol% are ratios in which changes are small, while TM is a value, and its change is large. The levels of PTCA, PDCA, PTeCA, and TTCA had more or less positive correlations with *V* (Figure S7). It was reasonable that these levels increased with melanosome volume. Both levels of PTCA (*R*^2^ = 0.25, *p* = 0.0032) and PDCA (*R*^2^ = 0.23, *p* = 0.0068) showed significant correlations with age (Figure S7a,b), while TTCA level did not (*R*^2^ = 0.065, *p* = 0.21) (Figure S7d). Therefore, the increase of TM in relation to the increase of *V* could be ascribed to the increases of eumelanin. It should be noted that eumelanin is much darker than pheomelanin [15].

Considering the chemical reaction kinetics, it has been proposed that pheomelanic pigment is produced first in the process of mixed melanogenesis followed by the deposit of eumelanic pigment [23]. In theory, the pheomelanin mol% depends on the concentration of cysteine in melanosomes during melanogenesis and does not depend on the size of melanosomes. In the case of eumelanin, both levels of PTCA and PDCA significantly increased with *V,* but DHI mol% relating to their ratio had no correlation with *V*. For DHICA melanogenesis, the contribution of dopachrome tautomerase or copper ion (II) is known [21,24,25], and the concentration and the activity of such components during melanogenesis may be key factors for the DHI mol%. 

## 3. Discussion

### 3.1. Impacts of the Increase in PDCA by Aging

**Figure 6:**
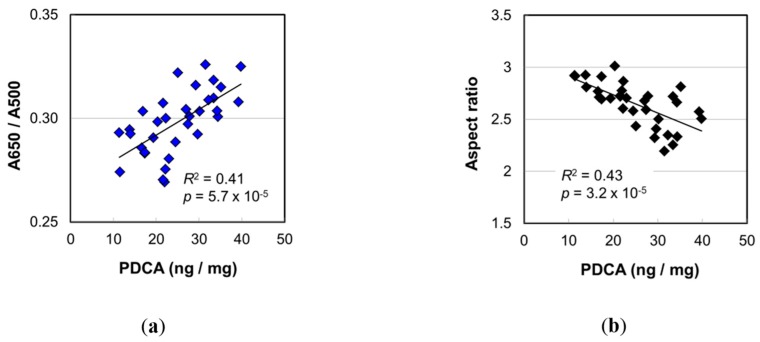
The contribution of PDCA level on (**a**) the absorbance ratio A650/A500 of Soluene-350 solution of hair melanin and (**b**) the aspect ratio of the isolated melanosome. The coefficient of determination and the *p*-value are shown in each figure.

The PDCA level, a marker of DHI melanin, showed the highest correlation with age among the values determined in the melanin analyses (Figure 3). To examine the effect of PDCA level, we performed the correlation analyses of the PDCA level with various values determined in this study. Figure 6a,b show the plots of A650/A500 and the mean aspect ratio of isolated melanosomes, respectively, against PDCA level. Both showed very high significance (*R*^2^ = 0.41, *p* = 5.7 × 10^−5^ for A650/A500; *R*^2^ = 0.43, *p* = 3.2 × 10^−5^ for aspect ratio).

The A650/A500 of the melanin solution in Soluene-350 also showed a significant correlation with age (Figure 3b) (*R*^2^ = 0.23, *p* = 0.0050). This meant there was a change in color shade of the solubilized hair melanin. The cause of the color shade change was presumed to be the transformation of the ratio of pigment components. It is known that eumelanin and pheomelanin have different colors—black-brown and yellow-reddish brown, respectively. In this study, however, it was found that pheomelanin mol% was independent of age (Figure 4b). Regarding the component of eumelanin, we found that the DHI mol% significantly increased with age (*R*^2^ = 0.31, *p* = 0.00072). The high correlation between A650/A500 and the PDCA level (Figure 6a) suggested the contribution of DHI melanin content to the A650/A500. A weak correlation of A650/A500 was also found with DHI mol% (Figure S8a) (*R*^2^ = 0.12, *p* = 0.051).

The a^*^ and b^*^ values decreased with age for actual hairs (Figure 1). However, in the case of dark colored hair, the decreases in a^*^ and b^*^ may have been caused by the fact that the chromatic value decreased as the hair color became darker (L^*^ decreased). In order to examine the change in hair color shade, we calculated the Metric Hue-Angle *h* (=tan^−1^(b^*^/a^*^)), but we could not find the age dependence of *h* nor a significant correlation of *h* with A650/A500 (Figure S9). The reason for there being no significant relationship between the actual hair color shade and the spectrum of the extracted melanin may have been attributed to the light scattering due to micro pores existing at the hair surface or inside the hair. The micro pores may have led to ambiguous hair color shade [26].

**Figure 7:**
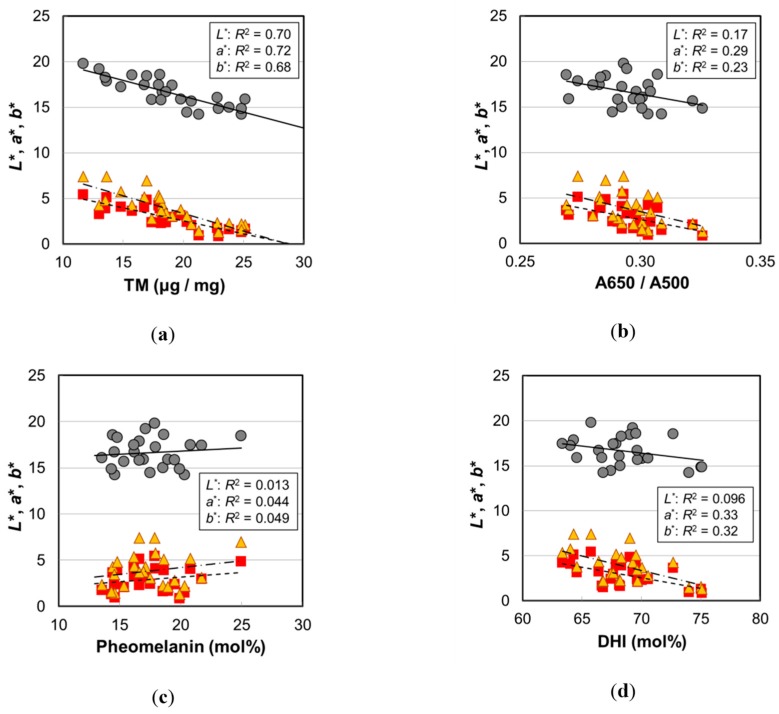
The hair color parameters (L^*^, a^*^, and b^*^) of the Japanese female hair samples plotted against various values measured by hair melanin analyses. Circles, L^*^; squares, a^*^; triangles, b^*^. Lines are the fitted curves. Solid lines, L^*^; dashed lines, a^*^; dot-and-dash lines, b^*^. (**a**) The color values vs. total melanin amount, *p* = 2.2 × 10^−7^ (L^*^), 1.0 × 10^−7^ (a^*^), and 3.9 × 10^−7^ (b^*^); (**b**) vs. A650/A500, *p* = 0.043 (L^*^), 0.0054 (a^*^), and 0.015 (b^*^); (**c**) vs. pheomelanin mol%, *p* = 0.59 (L^*^), 0.31 (a^*^), and 0.29 (b^*^); (**d**) vs. DHI mol%, *p* = 0.13 (L^*^), 0.0027 (a^*^), and 0.0032 (b^*^). The coefficients of determination are shown in each figure.

Panzella et al. [10] examined the structures of synthesized DHI melanin and DHICA melanin prepared by oxidation from DHI and DHICA. According to them, DHI melanin formed small globular aggregations (<100 nm), and DHICA melanin formed rod-like assemblies (ca. 1 μm) of smaller elongated aggregates (>100 nm). It was noted that the size of assemblies of DHICA eumelanin was comparable to that of melanosomes. From the correlation analyses between melanin marker levels and melanosome morphology parameters, we found the highest correlation between the PDCA level and the aspect ratio of melanosome (Figure 7b). The significance was low, however, and the DHI mol% was also found to be significantly correlated with the aspect ratio (Figure S8b) (*R*^2^ = 0.21, *p* = 0.0072). It can be said that the more the DHI melanin there was, the smaller the aspect ratio of melanosome was—that is, the shape of melanosome showed a trend of changing from a slender, ellipsoidal shape to a thick shape. This trend was consistent with the results of Panzella et al. [10]. Under the consideration that the shape of melanosome is not fixed when melanin is synthesized in a living cell and that the shape could be affected by the materials formed inside the cell, it is possible that the stacking of assemblies of melanin molecules influences the melanosome morphology. Increase in DHI mol% may disturb the elongated aggregates of DHICA melanin. In principle, if a surface area is the same, the internal volume is larger for a round shape than an ellipsoidal shape. Therefore, in response to an increase in eumelanin, particularly DHI melanin, the melanosome shape may change to be thicker. From the above, the increase in DHI melanin seems to be one of the causes of the shape of melanosomes becoming thicker by aging.

### 3.2. Causes of Hair Color Darkening by Aging

In order to examine the correlation with hair color, various melanin parameters are plotted against L^*^, a^*^, and b^*^ in Figure 7, and *p*-values of the correlations are summarized in Table 1. All the color parameters (L^*^, a^*^, and b^*^) showed very high correlations with TM (Figure 7a). The decrease in L^*^ meant darker color, and the decreases in a^*^ and b^*^ indicated the change toward achromatic. In addition, although the correlations were not so high, they showed significant correlations with A650/A500 (Figure 7b). The color of pheomelanin is yellow to reddish-brown and is expected to affect hair color shade, but there was no correlation between the pheomelanin mol% and L^*^, a^*^, and b^*^ values (Figure 7c). In contrast, DHI mol% showed significant correlations with a^*^ and b^*^ (Figure 7d), suggesting a potential contribution to the hair color shade.

The high correlation of TM with L^*^ was very reasonable from the perspective that melanin is the component that gives color to the hair, which was certainly verified by this result. The L^*^ value also showed a high correlation with age; however, there was a variation due to individual differences (Figure 1). In contrast, the data points of TM had less variation (Figure 7a). It is likely that TM is the parameter that reflects the hair color more directly.

The absorbance of DHI melanin was larger compared to DHICA melanin (Figure 2a and Figure S6a). That is, when the DHI mol% increased, the hair became darker. Therefore, the increase in DHI mol% by aging may have contributed to the hair color darkening; however, the correlation between the DHI mol% and L^*^ was not significant (*p* = 0.13) (Table 1). This seems to be attributed to the fact that the contribution of TM to the hair darkening is dominant.

The factors attributed to TM should be the number of melanosome per unit volume of hair, the volume of melanosome, and the density of melanin inside melanosome. Among these, the melanosome number per cross-sectional cortex area was estimated in the previous study [13], and it was found that the melanosome number was independent of age. In contrast, according to the fact that the volume of melanosome significantly correlates with age, the increase in TM could be attributed to the increase in melanosome volume. Conversely, it might be said that the melanosome grows with an increase in melanin amount.

It is difficult to directly evaluate the melanin density in melanosome. However, we can estimate it using the results obtained in this study with the following equation:

*ρ*_M_ = TM · *ρ*/(*V*·*N*) ~ Const. · (TM/*V*) (1)

Here, *ρ*_M_ is the melanin density inside melanosome, *ρ* is the hair density, and *N* is the number of melanosome per unit volume of hair. *N* has no age dependence, and *ρ* seems to be almost constant, though its age dependence is unknown. Then, we calculated TM/*V* values and plotted against age (Figure S10). As the result, no age dependence was seen and, consequently, it could be said that the increase in TM with age was mainly caused by the enlargement of melanosome size.

The distribution of the melanin pigment may also affect the hair color. In regard to this point, we performed optical microscopy on hair cross sections of 10 μm thickness. Per the results, we found that melanin pigments were distributed uniformly or presented in the outer periphery of the cortex, but there was no apparent change with age (Figure S11).

In conclusion, hair color darkening by aging is most affected by the increase in TM relating to the enlargement of the melanosome size, followed by the increase in DHI melanin ratio to the eumelanin in the hair.

## 4. Materials and Methods

### 4.1. Hair Samples

All the hair samples used in this study were non-chemically treated hairs and were provided by Japanese female volunteers with consent regarding the use of hair samples for the research. The root side part of the hair fibers (as much as possible) was used for the experiments. It was unclear which part was cut before it was provided, and the hair samples may have been somewhat affected by weathering and repeated shampooing. In the case that the hair samples included some gray hairs, we removed the gray hair fibers and used only pigmented hairs. Hairs were used without any particular washing.

### 4.2. Chemicals

For the isolation of melanosome from hair, the following chemicals were used: Proteinase K (21 U/mg) (FUJIFILM Wako Pure Chemical Corp., Osaka, Japan), Papane (MP Biomedicals, LLC, Santa Ana, USA), Protease (38.4 U/mg) (FUJIFILM Wako Pure Chemical Corp., Osaka, Japan), and dithiothreitol (for molecular biology, FUJIFILM Wako Pure Chemical Corp., Osaka, Japan). All other chemicals were of the purest grade commercially available.

For melanin synthesis and analysis, the following chemicals were used. Tyrosinase (from mushrooms, specific activity 1715 U/mg) was purchased from Sigma-Aldrich (St. Louis, MO, USA). DHI and DHICA were prepared as described in d’Ischia et al. [17]. Soluene-350 was purchased from Perkin-Elmer (Waltham, MA, USA). The 6 M HCl and 57% HI were purchased from Wako Pure Chemicals (Osaka, Japan). All other chemicals were of the purest grade commercially available.

### 4.3. Hair Diameter

Hair samples were left in a room controlled at 20 °C and 65% relative humidity overnight and then used for the diameter measurement in the same room. Hair diameters were measured for 10 hair fibers selected randomly from each subject with a laser scan micrometer (LSM-500S; Mitutoyo Corp., Kawasaki, Japan). The orthogonal projection of the hair fiber was measured while it rotated. The minimum and the maximum values were evaluated, and the cross-sectional area was calculated assuming the hair cross section as an ellipsoid.

### 4.4. Colorimetric Measurement

Hair color was expressed by the parameters L^*^, a^*^, and b^*^ values in the standard CIEL^*^a^*^b^*^ color space defined by the International Commission on Illumination [27]. L^*^ represented the level of gray from black (L^*^ = 0) to white (L^*^ = 100), and a^*^ and b^*^ represented the red-green and the yellow-blue components, respectively. Positive values of a^*^ and b^*^ meant red and yellow colors, respectively. We evaluated L^*^, a^*^, and b^*^ values for 25 possible hair samples using a chroma meter (CR-400; Konica Minolta, Tokyo, Japan) with the illuminant D65. The measurements were performed at a minimum of five different locations on each hair bundle, and the average values were determined.

### 4.5. Isolation of Melanosomes and Morphology Measurement

We isolated melanosomes from about 10–15 mg of hair in mild conditions using three kinds of enzymes [13,28]. Next, we analyzed the morphological parameters of the isolated melanosomes in the same manner as described in the previous paper [13]. Dispersions of isolated melanosomes were filtered with suction on a membrane filter with 0.1 μm pore size (Isopore®; Merck KGaA, Darmstadt, Germany), the filtered melanosomes were observed by SEM (JSM-6330F; JEOL, Tokyo, Japan), and digital images were obtained. In order to have a statistical discussion about the size, the major and the minor axes of more than 100 melanosomes were measured using Image J software [29] with the digital images of SEM.

### 4.6. Melanin Analyses

Aqueous suspensions of the hair samples were prepared by homogenizing about 15 mg of hair in water at a concentration of 10 mg/mL with a Ten-Broeck glass homogenizer. Aliquots of 100 μL (1 mg) were solubilized in Soluene-350 solution, and absorbances at 500 nm and 650 nm, A500 and A650, respectively, were measured [15]. In the analyses, background absorbances of albino hair (A500 = 0.014, A650 = 0.001) were subtracted. From the value of A500, total melanin amount TM was determined.

According to the method of Ito et al. [16], levels of PTCA, PDCA, thiazole-2,4,5-tricarboxylic acid (TTCA), and PTeCA were measured by alkaline H_2_O_2_ oxidation. Next, HI reductive-hydrolysis was performed to measure 4-amino-3-hydroxyphenylalanine (4-AHP) level, as described in Wakamatsu, Ito, and Rees [30]. Then, according to the method by Ito et al. [12], aqueous suspensions of 200 μL (2 mg) were subjected to acid hydrolysis with 6M HCl followed by alkaline H_2_O_2_ hydrolysis to measure levels of PTCA, PDCA, PTeCA, and TDCA. It was found that the TDCA/PDCA ratio had a linear correlation with pheomelanin mol% [12]. We used the relation as a calibration curve to determine pheomelanin mol% in the extracted hair melanins (Figure S12).

### 4.7. Preparation of Synthetic Melanins and Analyses

Mixtures of DHI and DHICA with various mixing ratios were oxidized by tyrosinase to prepare synthetic melanins, as described in d’Ischia et al. [17]. One hundred µL of 1 mg/mL suspensions (0.1 mg) of the synthetic melanins were oxidized by alkaline H_2_O_2_ as above to measure PTCA and PDCA levels. With the obtained relationship between the ratio of PDCA/PTCA and the DHI mol% of the mixture, DHI mol% values in eumelanin of the hair samples were determined (Figure S13) [31]. 

### 4.8. Statistical Analyses

Pearson’s product-moment correlation test was used. Linear regression analysis was employed to determine the coefficient of determination *R*^2^ and *p*-value for the slope coefficient with Microsoft Excel.

## 5. Conclusions

In this study, it was possible to examine the effect of aging on hair melanin and melanosomes using pigmented hairs collected from subjects in a very wide range of age. As the result, it was found that hair color darkening by aging is most affected by the increase in TM relating to the enlargement of the melanosome size, followed by the increase in DHI melanin ratio to the eumelanin in the hair. In addition, it was suggested that the increase in DHI melanin by aging contributes to the change in melanosome morphology.

One advantage of using human hair in the study is the ease of sampling. Even for human skin, it is known that the skin color changes with age [32]. The analysis of skin melanin was performed previously [33], however, studies in a wide age range have not been reported yet. The age change in melanins and melanosomes of skin is of interest as a further study. In particular, the proportion of DHICA affects the free-radical-scavenging ability; hence, it would be worth examining the age dependence of melanin composition for considering the mechanism of skin aging.

## Figures and Tables

**Table 1:** *p*-values by the regression analysis between hair color parameters and various values.

Parameter		Hair Color		
		*L* ^*^	*a* ^*^	*b* ^*^
Human	Age (year)	3.3 × 10^−5^	1.1 × 10^−6^	5.6 × 10^−6^
Fiber	Cross sectional area (μm^2^)	0.63	0.28	0.12
Melanosome	Major axis (μm)	0.72	0.26	0.37
	Minor axis (μm)	0.0063	0.0043	0.0024
	Aspect ratio	4.1 × 10^−5^	1.2 × 10^−5^	1.5 × 10^−5^
	Volume (μm^3^)	0.011	0.066	0.038
Melanin	Total melanin amount (μg/mg)	2.2 × 10^−7^	1.0 × 10^−7^	3.9 × 10^−7^
	A650/A500 ^1^	0.043	0.0054	0.015
	DHI ^2^ (mol%)	0.13	0.0027	0.0032
	Pheomelanin (mol%)	0.59	0.31	0.29
	PTCA ^3^ level (ng/mg)	1.2 × 10^−6^	9.4 × 10^−7^	1.7 × 10^−6^
	PDCA ^4^ level (ng/mg)	8.6 × 10^−7^	1.8 × 10^−10^	1.5 × 10^−9^
	PTeCA ^5^ level (ng/mg)	0.00087	0.0094	0.0057

^1^ The ratio of absorbance at the wavelength of 650 nm (A650) and 500 nm (A500), ^2^ 5,6-dihydroxyindole, ^3^ pyrrole-2,3,5-tricarboxylic acid, ^4^ pyrrole-2,3-dicarboxylic acid, ^5^ pyrrole-2,3,4,5-tetracarboxylic acid.

## Supplementary Materials

Supplementary materials can be found at https://www.mdpi.com/1422-0067/20/15/3739/s1.

## Abbreviations

A500	Absorbance at the wave length of 500 nm
A650	Absorbance at the wave length of 650 nm
4-AHP	4-Amino-3-hydroxyphenylalanine
DHI	5,6-Dihydroxyindole
DHICA	5,6-Dihydroxyindole-2-carboxylic acid
H_2_O_2_	Hydrogen peroxide
HPLC	High performance liquid chromatography
PDCA	Pyrrole-2,3-dicarboxylic acid
PTCA	Pyrrole-2,3,5-tricarboxylic acid
PTeCA	Pyrrole-2,3,4,5-tetracarboxylic acid
TDCA	Thiazole-4,5-dicarboxylic acid
TM	Total melanin amount
TTCA	Thiazole-2,4,5-tricarboxylic acid
UV	Ultraviolet
