# Supplementary material for: Effects of Aging on Hair Color, Melanosome Morphology, and Melanin Composition in Japanese Females
Source: Int J Mol Sci. 2019 Jul 31;20(15):3739. doi: 10.3390/ijms20153739 (PMC6696507; doi:10.3390/ijms20153739)
Supplement: Supplementary file 1 [file ijms-20-03739-s001.pdf]

Supporting Information

Effects of aging on hair color, melanosome morphology, and melanin composition in Japanese females

Takashi Itou [1], Shosuke Ito [2] and Kazumasa Wakamatsu [2]

[1]Kao Corporation, R&D Development Research - Hair Care Products Research, 2-1-3, Bunka, Sumida-ku, Tokyo 131-8501, Japan

[2]Department of Chemistry, Fujita Health University School of Medical Sciences, 1-98, Dengakugakubo, Kutsukake-cho, Toyoake, Aichi 470-1192, Japan

Table S1. The hair samples and the determined values of hair color, fiber cross-sectional area, and major and minor axis of the isolated melanosomes

Sample name	Age/ year old	Hair tress CIELAB coordinates			Fiber Cross-sectional area (μm^2)	Melanosome		
		$L*$	$a*$	$b*$		[1]n	Mean ± SD (μm) Major axis	Minor axis
SA	4	16.7	3.2	4.3	4038	126	0.80 ± 0.14	0.28 ± 0.05
002	5	17.8	5.1	7.4	4058	139	0.90 ± 0.14	0.32 ± 0.06
020	6	19.8	5.4	7.4	4188	118	0.80 ± 0.12	0.28 ± 0.06
022	6	17.5	4.1	5.2	4707	170	0.89 ± 0.14	0.31 ± 0.05
MM	10	18.4	4.8	7.0	5465	142	0.89 ± 0.17	0.34 ± 0.07
HD5-42	11	17.2	4.1	5.7	7034	233	0.87 ± 0.16	0.32 ± 0.07
MO	14	19.2	3.3	4.3	4652	149	0.85 ± 0.12	0.30 ± 0.06
E	14	15.9	3.2	3.8	5877	192	1.00 ± 0.23	0.40 ± 0.14
MY	18	-	-	-	-	141	0.91 ± 0.21	0.34 ± 0.09
SD	21	17.4	2.9	3.1	8013	188	0.87 ± 0.19	0.34 ± 0.08
B20	23	18.5	3.6	4.3	8162	154	0.95 ± 0.21	0.35 ± 0.07
B05	24	-	-	-	-	123	0.79 ± 0.15	0.32 ± 0.06
B06	24	-	-	-	-	147	0.89 ± 0.15	0.33 ± 0.06
HD202	24	17.5	4.2	5.3	5747	128	0.89 ± 0.16	0.34 ± 0.08
053	31	16.1	1.7	2.3	6156	147	0.89 ± 0.17	0.34 ± 0.08
C07	34	-	-	-	-	118	0.84 ± 0.15	0.31 ± 0.08
C09	34	-	-	-	-	107	0.76 ± 0.16	0.32 ± 0.06
HD01	35	15.0	1.6	2.2	7071	167	0.77 ± 0.14	0.34 ± 0.08
050	36	18.6	3.9	5.1	6071	258	0.79 ± 0.17	0.31 ± 0.08
043	39	15.7	2.1	2.2	6758	177	0.82 ± 0.15	0.35 ± 0.09
042	40	18.3	3.9	4.8	5439	150	0.82 ± 0.13	0.31 ± 0.06
D06	41	-	-	-	-	149	0.76 ± 0.13	0.30 ± 0.07
D09	43	-	-	-	-	129	0.81 ± 0.15	0.33 ± 0.08
NF	43	15.9	1.7	2.1	8336	206	0.83 ± 0.16	0.33 ± 0.08
D02	44					115	0.83 ± 0.16	0.33 ± 0.07
HD131	46	16.7	2.4	3.5	4824	176	0.87 ± 0.17	0.34 ± 0.07
HD33	51	15.9	2.4	2.8	6089	184	0.86 ± 0.19	0.33 ± 0.08
ST	52	-	-	-	-	128	0.96 ± 0.18	0.40 ± 0.10
E01	53	-	-	-	-	163	0.94 ± 0.16	0.36 ± 0.09
HD75	55	14.2	1.5	2.2	4817	220	0.82 ± 0.15	0.36 ± 0.08
SC	56	14.5	2.4	3.0	5575	133	0.91 ± 0.17	0.37 ± 0.08
MU	57	15.8	2.3	3.7	5981	111	0.86 ± 0.16	0.31 ± 0.06
HD40	62	14.9	0.9	1.3	4527	171	0.84 ± 0.14	0.39 ± 0.07
HD24	65	14.2	0.9	1.4	5833	145	0.82 ± 0.15	0.34 ± 0.08
HD22	67	-	-	-	-	127	0.84 ± 0.14	0.38 ± 0.10
HD26	68	14.9	1.3	1.6	5422	159	0.85 ± 0.16	0.38 ± 0.08
KH	72	-	-	-	-	170	0.84 ± 0.16	0.37 ± 0.09
YN	75	-	-	-	-	113	0.91 ± 0.15	0.40 ± 0.08

[1]n, the number of the isolated melanosomes used in the size measurements.

Table S2. Levels of various melanin markers in the hair samples

Sample name	Age (years old)	Soluene-350 Solubilization		H_2O_2 oxidation (ng/mg)				H_2O_2 oxidation after HCl hydrolysis (ng/mg)				4-AHP (ng/mg)	TM (µg/mg)	Pheo-melanin (mol%)	DHI (mol%)
		A500 (mg^{-1})	A650/A500 ratio	PTCA level	PDCA level	TTCA level	PTeCA level	PTCA level	PDCA level	TDCA level	PTeCA level				
SA	4	0.181	0.298	216	20.4	39.1	68.7	90.5	11.1	37.5	68.5	4.0	18.3	16.2	66.4
002	5	0.135	0.274	133	11.5	24.8	48.8	57.8	6.9	23.9	36.1	5.4	13.6	16.7	64.3
020	6	0.116	0.293	124	11.4	24.7	35.1	59.9	6.8	25.3	29.5	3.6	11.7	17.9	65.8
022	6	0.166	0.283	174	17.4	39.1	62.7	66.4	6.9	29.9	46.0	4.1	16.8	20.8	67.9
MM	10	0.168	0.286	159	16.6	54.8	66.3	46.8	5.1	26.5	34.2	3.7	17.0	25.0	69.1
HD5-42	11	0.147	0.293	164	14.0	31.9	55.1	75.7	8.0	29.9	51.2	4.4	14.8	18.0	64.0
MO	14	0.129	0.295	131	13.8	28.3	39.0	56.7	8.1	28.9	32.2	2.2	13.0	17.2	69.3
E	14	0.196	0.270	247	21.6	48.9	87.5	108	10.6	37.4	78.3	3.5	19.8	17.0	64.6
MY	18	0.223	0.318	266	33.4	58.3	81.7	122	16.3	44.0	74.7	6.8	22.5	13.0	74.6
SD	21	0.189	0.280	232	23.0	77.9	82.0	107	10.0	45.2	71.0	20.2	19.1	21.7	67.7
B20	23	0.156	0.269	186	22.0	35.0	52.5	66.8	10.0	30.1	43.7	3.4	15.8	14.5	72.7
B05	24	0.276	0.308	357	39.3	70.5	104	155	18.3	60.7	108	13.8	27.9	15.9	70.5
B06	24	-	-	-	-	-	-	-	-	-	-		-	-	-
HD202	24	0.178	0.303	204	16.9	46.1	75.4	111	10.3	34.7	60.5	3.5	18.0	16.2	63.4
053	31	0.226	0.301	275	27.8	42.1	71.4	107	15.4	43.5	59.9	2.9	22.8	13.6	68.2
C07	34	0.2	0.315	279	35.2	64.3	87.5	110	13.1	48.1	69.5	9.5	20.2	17.7	74.8
C09	34	-	-	-	-	-	-	-	-	-	-		-	-	-
HD01	35	0.236	0.292	293	29.7	77.2	94.7	146	15.4	59.5	93.7	8.2	23.8	18.6	68.3
050	36	0.179	0.307	203	21.6	64.0	71.2	90.9	10.6	41.1	63.3	6.5	18.1	18.6	69.6
043	39	0.205	0.322	235	25.1	46.6	76.1	102	13.4	42.9	64.0	4.1	20.7	15.4	69.7
042	40	0.134	0.284	171	17.4	37.7	52.5	73.5	9.4	29.0	48.9	3.1	13.5	14.8	68.4
D06	41	0.138	0.275	176	22.2	58.9	46.7	70.4	9.7	40.5	45.3	25.7	13.9	20.1	74.8
D09	43	-	-	-	-	-	-	-	-	-	-		-	-	-
NF	43	0.249	0.297	287	27.4	74.2	97.1	128	14.0	55.3	94.5	4.6	25.1	19.0	66.7
D02	44	-	-	-	-	-	-	-	-	-	-		-	-	-
HD131	46	0.184	0.304	253	27.0	53.6	72.5	106	14.9	45.2	72.7	1.4	18.6	14.6	69.7
HD33	51	0.172	0.291	176	19.4	58.9	71.2	85.9	9.7	39.4	63.1	2.9	17.4	19.5	70.6
ST	52	0.32	0.325	432	39.8	82.6	86.6	144	23.7	70.2	100	0.7	32.3	14.2	65.8
E01	53	0.224	0.304	297	34.3	64.2	80.0	116	16.6	53.1	82.4	2.0	22.6	15.4	72.0
HD75	55	0.246	0.309	337	32.3	74.3	105	135	14.9	63.1	86.2	2.9	24.8	20.4	66.8
SC	56	0.201	0.289	249	24.5	42.1	83.4	107	12.2	44.5	62.8	6.3	20.3	17.5	67.5
MU	57	0.18	0.300	206	22.3	44.8	52.2	77.5	12.2	42.2	43.6	4.9	18.2	16.6	70.1
HD40	62	0.227	0.326	242	31.5	58.0	63.9	89.3	11.7	48.5	61.8	3.0	22.9	19.9	75.1
HD24	65	0.211	0.303	245	30.2	40.8	60.4	94.4	14.7	44.7	53.5	4.5	21.3	14.6	74.0
HD22	67	0.193	0.316	222	29.3	55.8	65.3	84.4	11.7	43.9	49.6	2.3	19.5	18.0	75.2
HD26	68	0.246	0.301	269	34.4	48.9	65.1	107	17.5	52.2	61.4	5.0	24.8	14.3	75.0
KH	72	-	-	-	-	-	-	-	-	-	-		-	-	-
YN	75	0.213	0.310	268	33.4	50.1	78.4	111	16.9	48.7	77.3	1.6	21.5	13.9	74.4

PTCA: pyrrole-2,3,5-tricarboxylic acid, PDCA: pyrrole-2,3-dicarboxylic acid, PTeCA: pyrrole-2,3,4,5-tetracarboxylic acid, TTCA: thiazole-2,4,5-tricarboxylic acid, TDCA: thiazole-4,5-dicarboxylic acid , 4-AHP: 4-amino-3-hydroxyphenylalanine

Figure S1

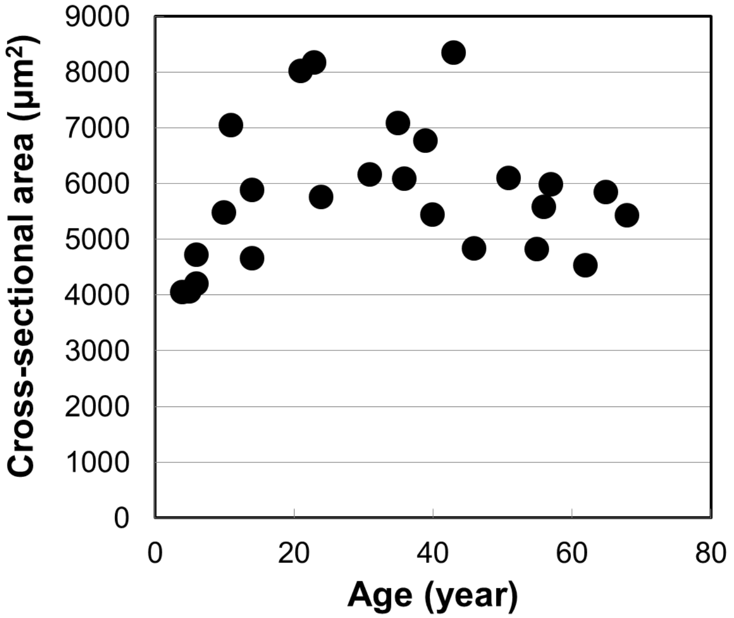

The age dependence of mean cross-sectional area of Japanese female hair samples used in this study.

Figure S2

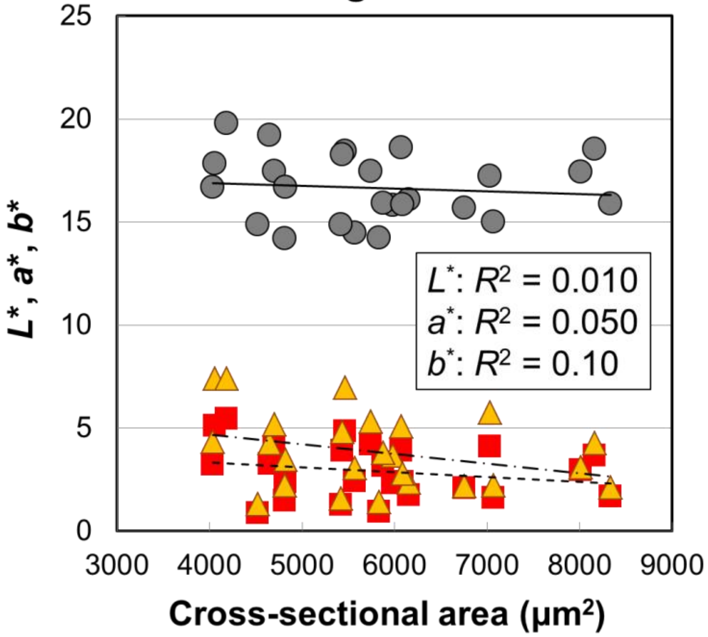

The L^*, a^*, and b^* values of the hair samples plotted against mean cross-sectional area of hair samples used in this study. $p = 0.63$ (L^*), 0.28 (a^*), and 0.12 (b^*).

Figure S3

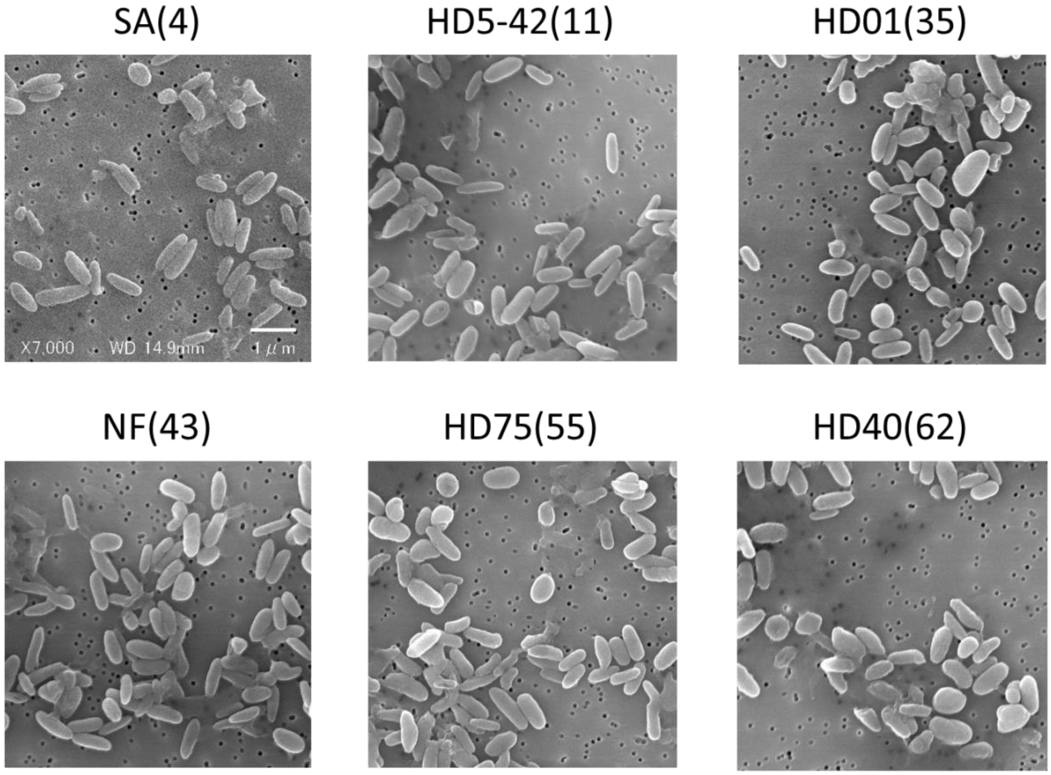

Figure S3 shows representative SEM images of melanosomes isolated from non-chemical-treated Japanese female pigmented hairs. Sample names are shown above the images. Numbers in parentheses are the age of the subjects. The scale bar in the figure of SA (upper and leftmost) means 1 μm and the magnification of all pictures are the same. Isolated melanosomes are ellipsoidal forms on the whole. Small dots with the dimension of 0.1μm are pores of the membranes.

Figure S4

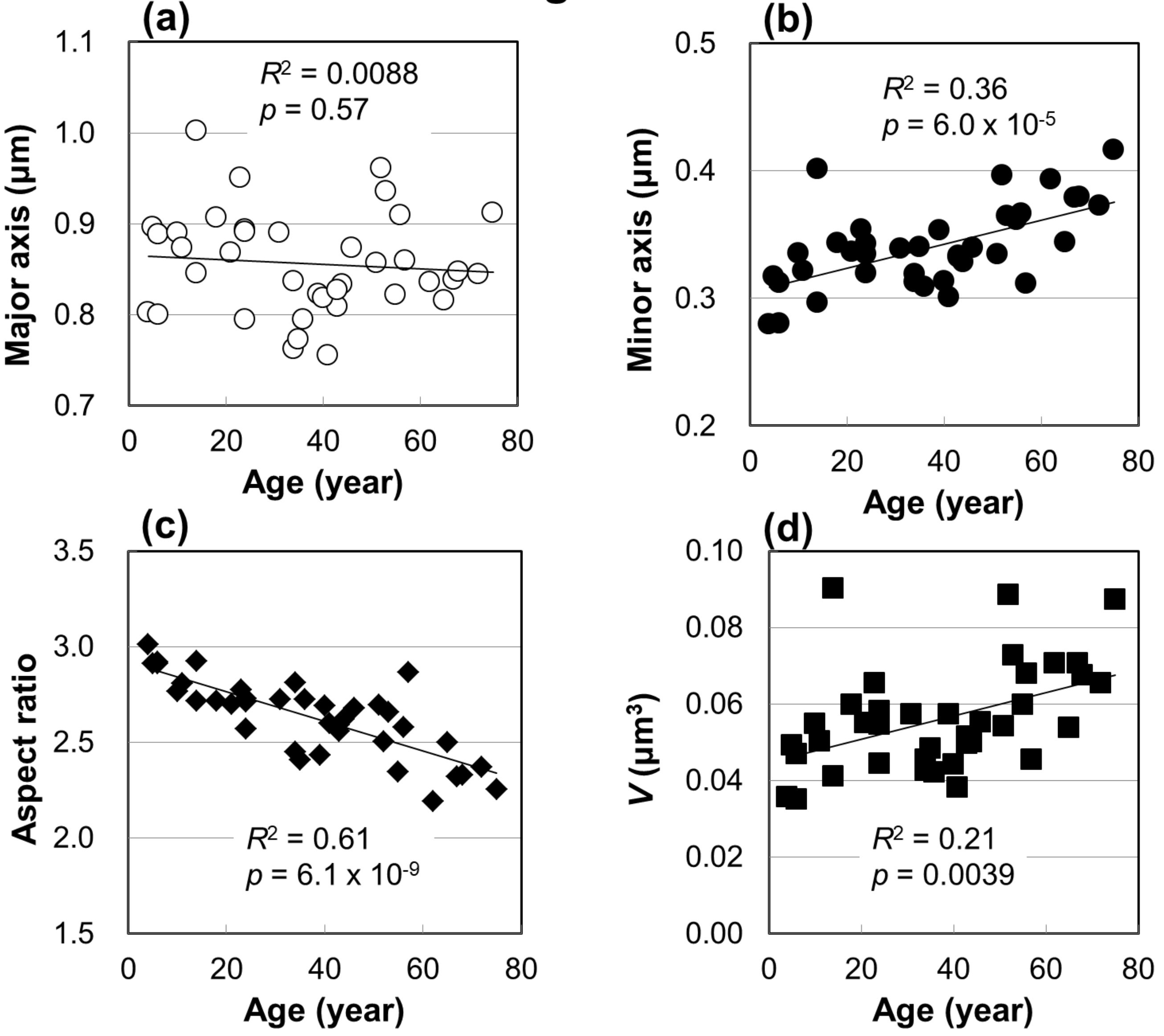

The age dependences of the morphological parameters determined for the malanosomes isolated from non-chemical treated Japanese female pigmented hairs in their age from 4 to 75. (a) Mean major axis. (b) Mean minor axis. (c) Mean aspect ratio. (d) Mean volume *V* with an assumption of ellipsoid.

Figure S5

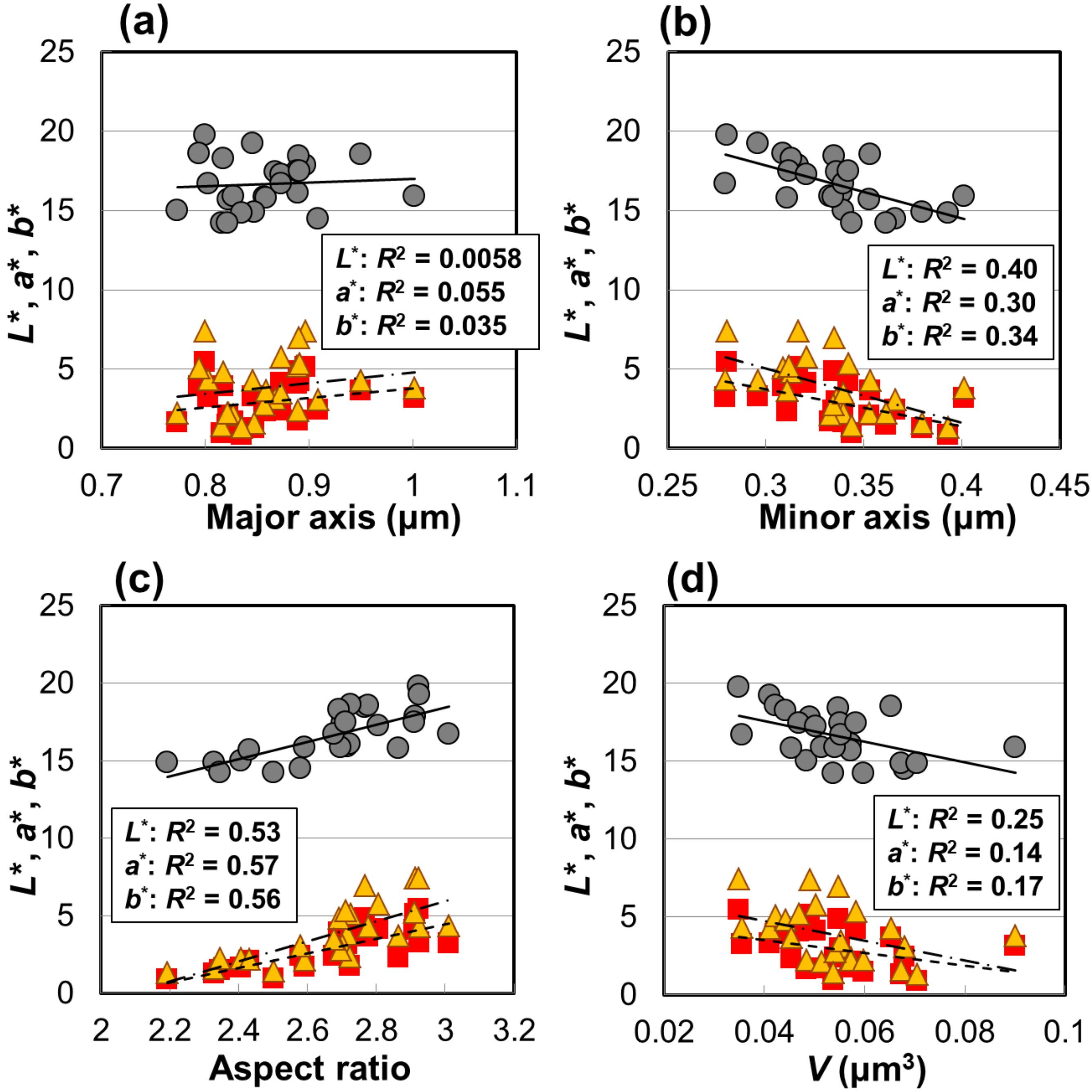

The L^*, a^*, and b^* values of the hair samples plotted against morphological parameters determined for the melanosomes isolated from non-chemical treated Japanese female pigmented hairs in their age from 4 to 68.

Figure S6

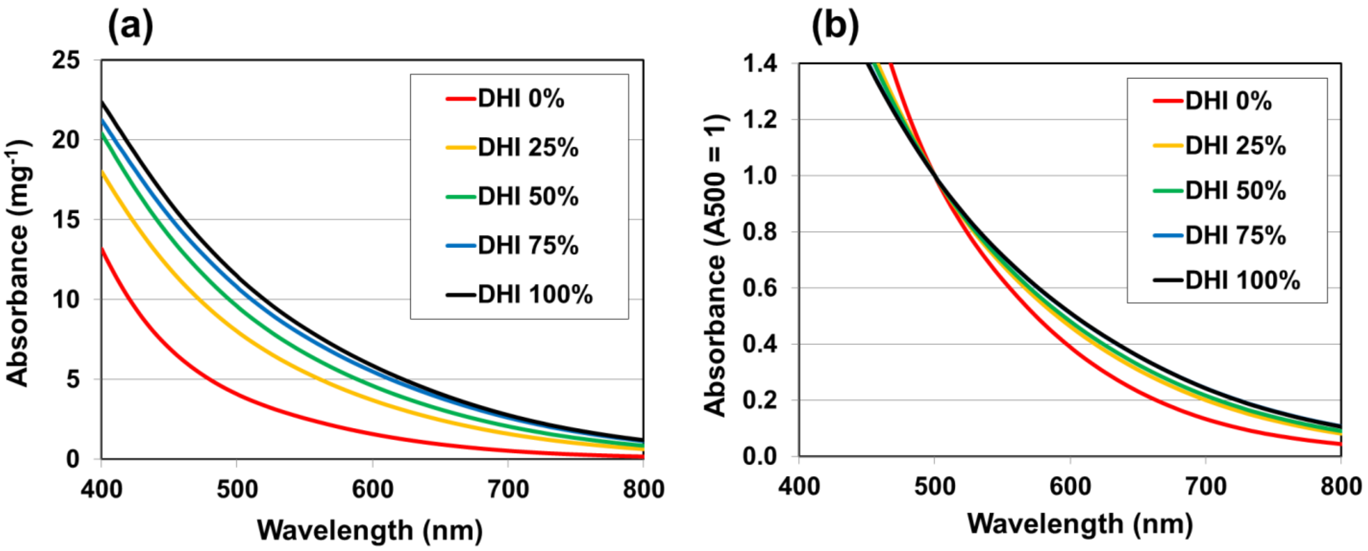

Absorption spectra of melanins synthesized from DHI and DHICA mixtures with various mol% of DHI. (a) Absorbance reduced by the mass of synthesized melanin. (b) Relative absorbance of synthesized melanin (A500 = 1).

Figure S7

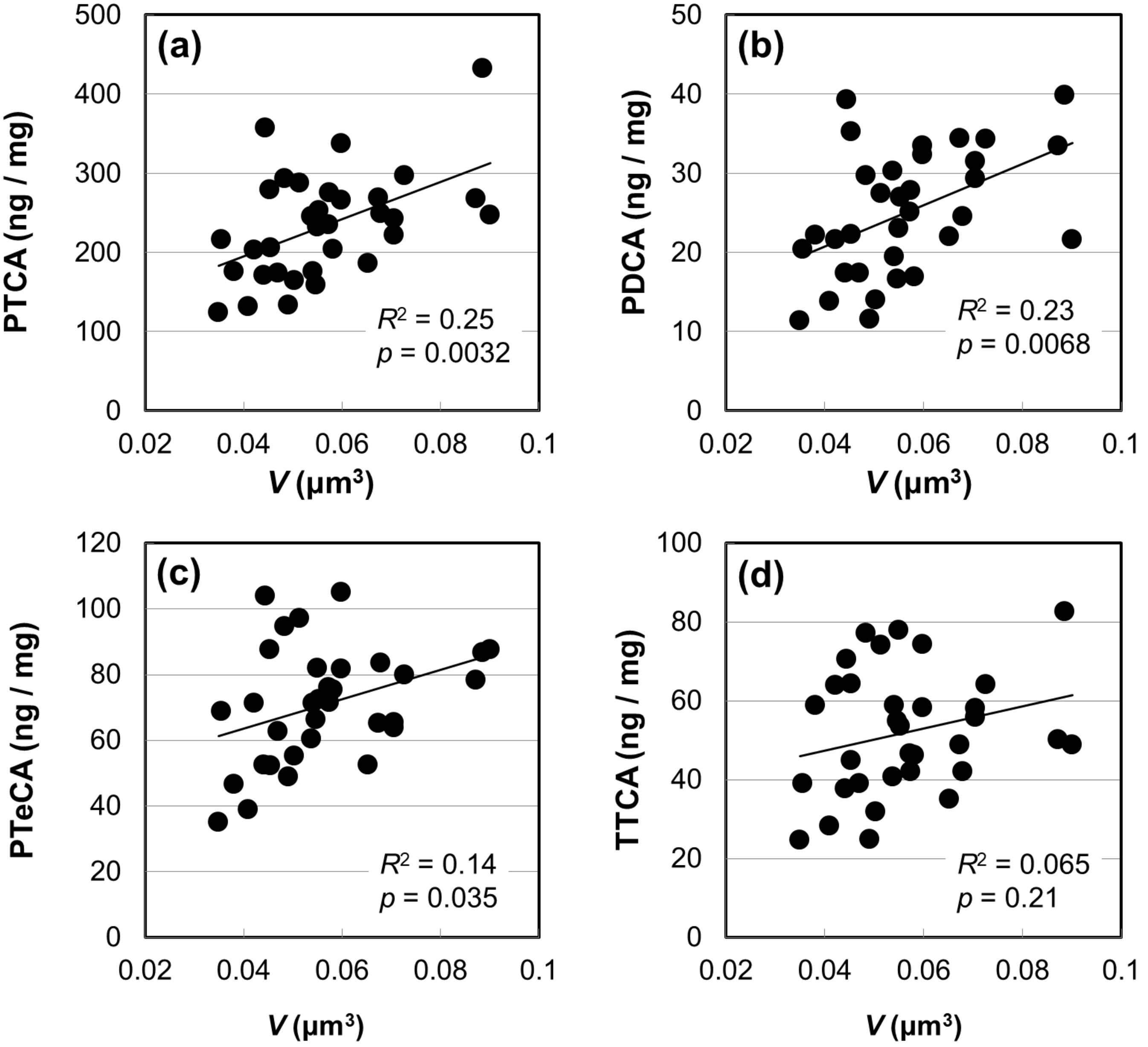

Correlations of various levels measured by hair melanin analyses vs. V. (a) PTCA, (b) PDCA, (c) PTeCA, (d) TTCA. As a whole, all levels have positive correlations with V. The levels of PTCA, PDCA, and PTeCA show significant correlations with V, while TTCA level does not.

Figure S8a

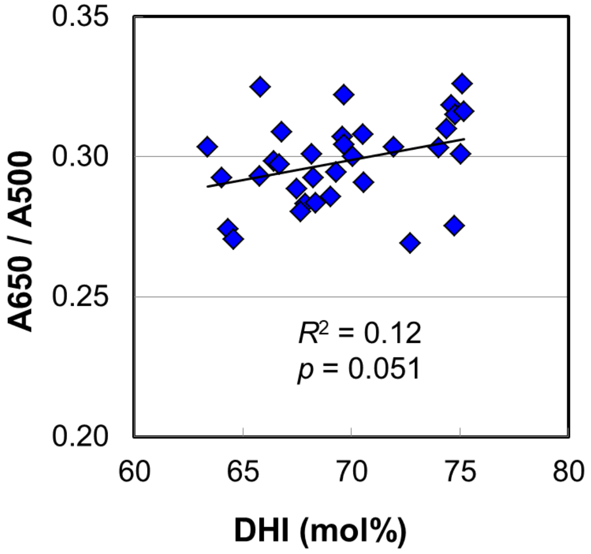

Figure S8b

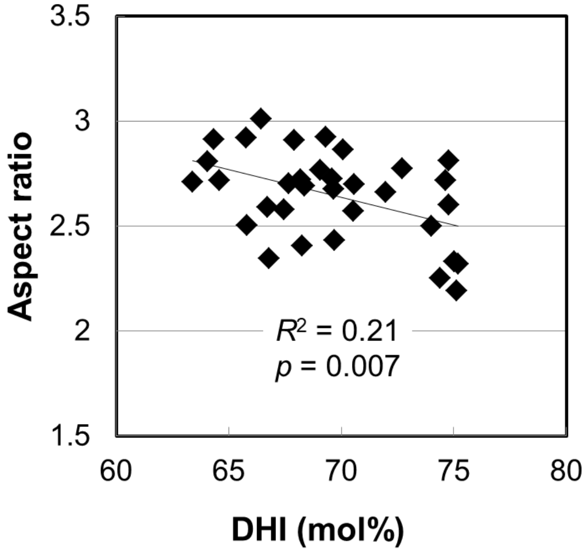

Figure S8a shows the contribution of DHI mol% on A650/A500 of Soluene-350 solution of hair melanin. This shows that DHI mol% has a weak positive correlation with A650/A500.

Figure S8b shows the contribution of DHI mol% to the aspect ratio of the isolated melanosome, suggesting that the aspect ratio significantly decreases with the increase in DHI mol%.

Figure S9a

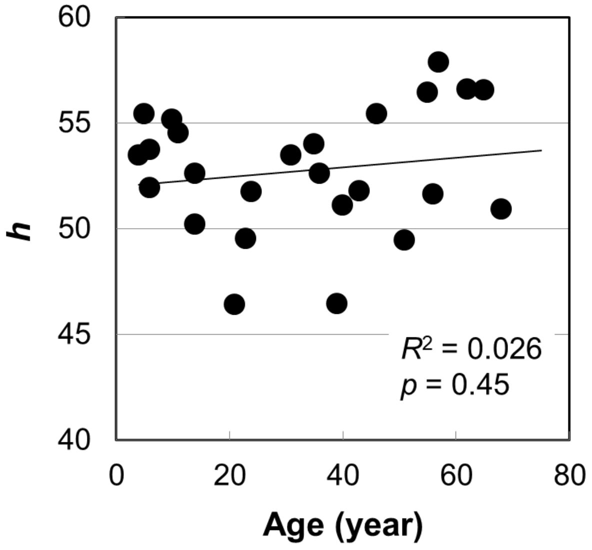

Figure S9b

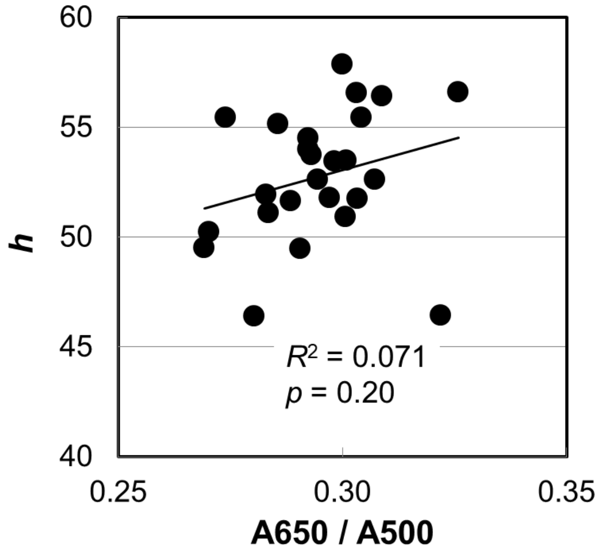

Figure S9a shows the age dependence of Metric Hue-Angle *h* determined from a^* and b^* values of hair bundles. There is no significant correlation.

Figure S9b shows the relationship between *h* and the ratio of absorbance ratio A650/A500 of Soluene-350 solution of hair melanin. There is no significant correlation.

Figure S10

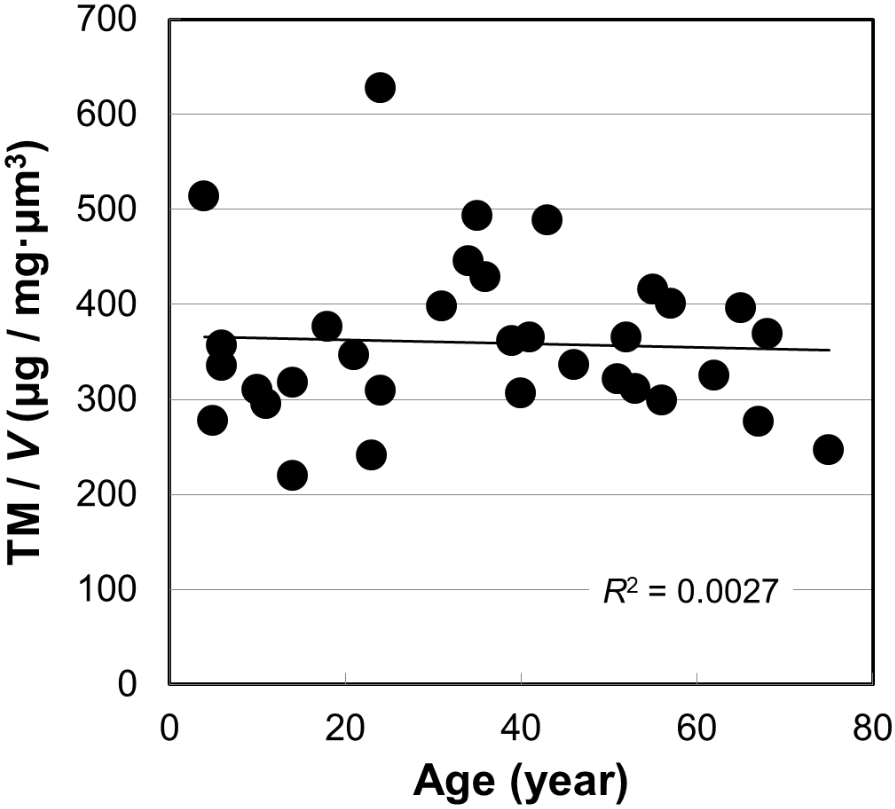

The age dependence of the evaluated parameter proportional to the melanin density in melanosomes.

Figure S11

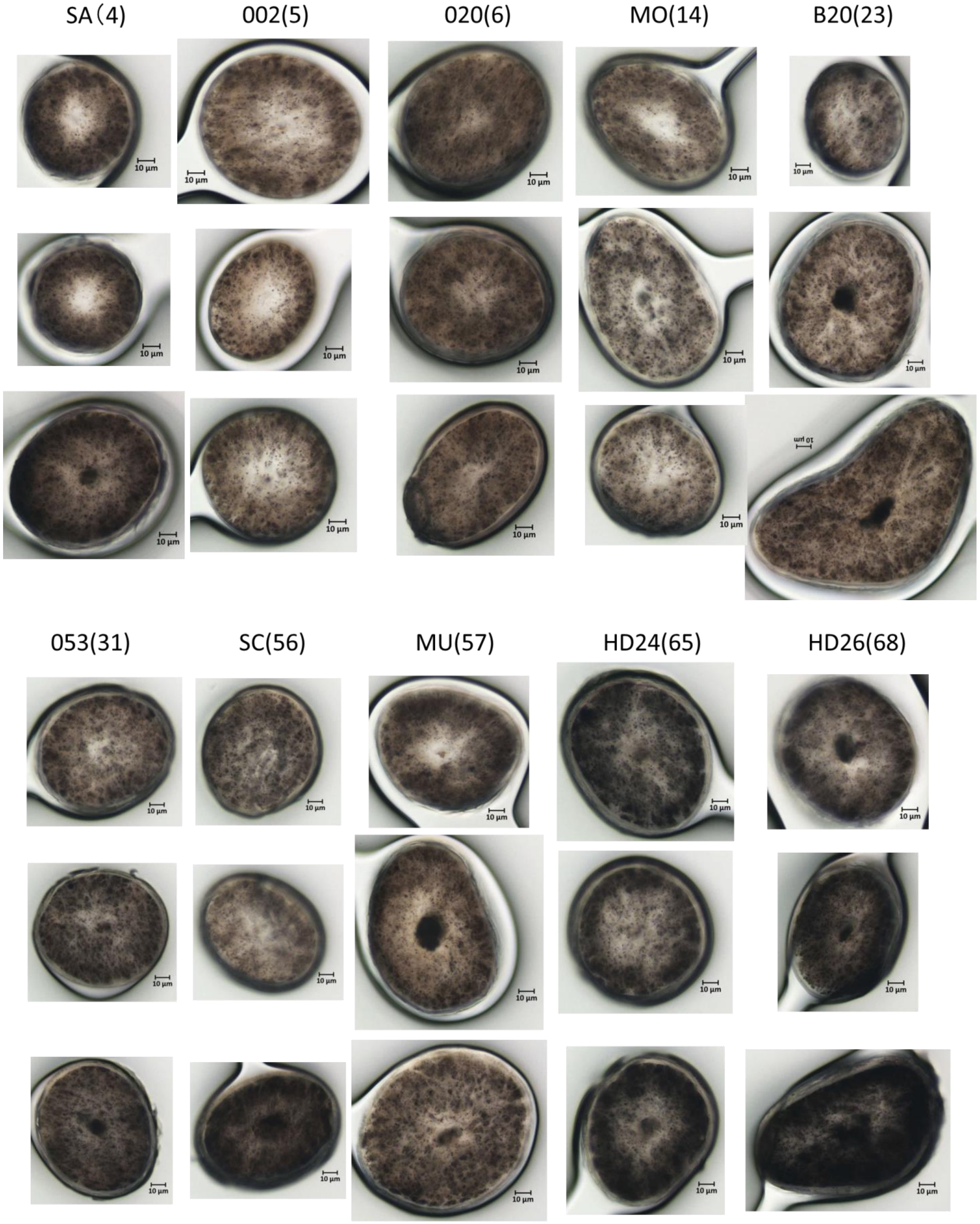

Figure S11 shows the optical microscope images of hair cross sections with the thickness of 10 μm for three hair fibers randomly selected from 10 subjects. Numbers in parenthesis are the age of the subjects. The distribution of melanin looks uniformly or periphery in hair cross-section and it has no age trend. It is noteworthy that the color shade of hairs over 60 looks more black.

Figure S12

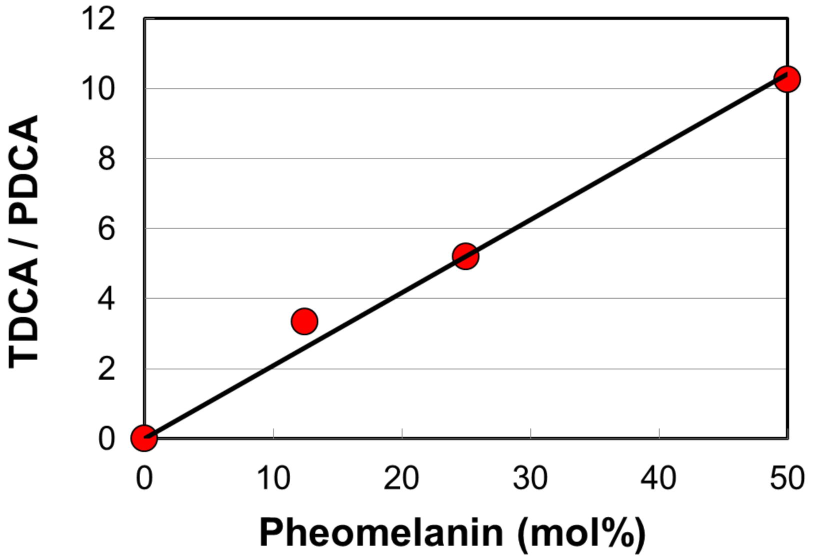

The calibration curve of TDCA/PDCA vs. Pheomelanin mol% in HCl-hydrolyzed melanins [12].

Figure S13

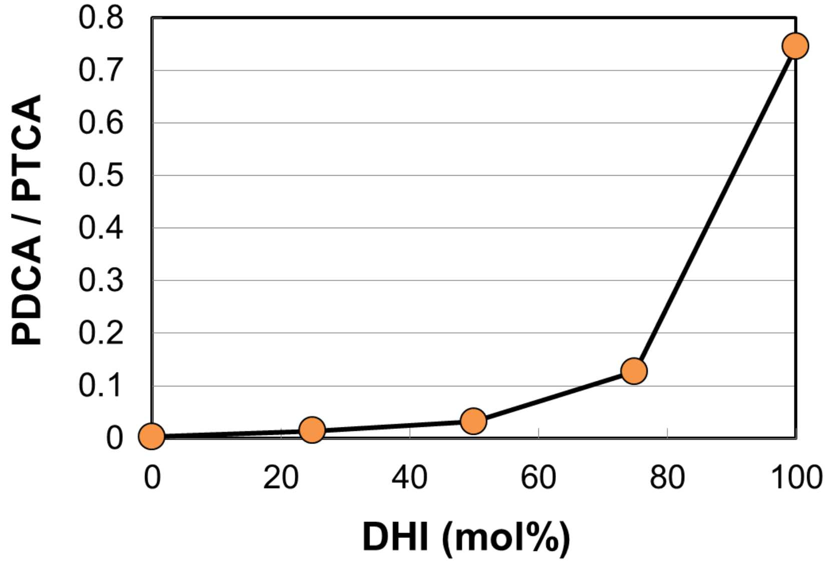

The calibration curve of PDCA/PTCA vs. DHI mol%.
